# Economic burden of antibiotic-not-susceptible isolates in uncomplicated urinary tract infection: Analysis of a US integrated delivery network database

**DOI:** 10.1186/s13756-022-01121-y

**Published:** 2022-06-14

**Authors:** Jason Shafrin, Alen Marijam, Ashish V. Joshi, Fanny S. Mitrani-Gold, Katie Everson, Rifat Tuly, Peter Rosenquist, Michael Gillam, Maria Elena Ruiz

**Affiliations:** 1 PRECISIONheor, Los Angeles, CA USA; 2 GlaxoSmithKline plc., Collegeville, PA USA; 3 PRECISIONheor, Austin, TX USA; 4 PRECISIONheor, Washington, DC USA; 5 MedStar Health, Washington, DC USA

**Keywords:** Urinary tract infection, Healthcare resource use, Costs, Antibiotic resistance

## Abstract

**Background:**

Uncomplicated urinary tract infections (uUTIs) are one of the most common bacterial infections in the United States (US). Contemporary data are important for understanding the health economic impact of antimicrobial-resistant uUTIs. We compared the economic burden among patients with uUTI isolates susceptible or not-susceptible to the initial antibiotic prescription.

**Methods:**

This retrospective cohort study utilized electronic health record data (1 July 2016–31 March 2020) from a large Mid-Atlantic US integrated delivery network database. Patients were females aged ≥ 12 years with a uUTI, who received oral antibiotic treatment and had ≥ 1 urine culture within ± 5 days of diagnosis. The primary outcome was the difference in healthcare resource use and costs (all-cause, urinary tract infection [UTI]-related) among patients with susceptible versus not-susceptible isolates during the 6 months after the index uUTI diagnosis. Secondary outcomes included: pharmacy costs, hospital admissions and emergency department visits, as well as the probability of uUTI progressing to complicated UTI (cUTI) between patients with susceptible and not-susceptible isolates. Patient outcomes were compared using 1:1 propensity score matching. Winsorized costs were adjusted to 2020 quarter 1 US dollars ($).

**Results:**

A total of 2565 patients were eligible for analysis. The propensity score-matched sample comprised 2018 patients, with an average age of 44.0 and 41.0 years for the susceptible and not-susceptible populations, respectively. In the 6 months post-index uUTI event, patients with not-susceptible isolates had significantly more all-cause prescriptions orders (+ 1.41 [*P* = 0.001]), UTI-related prescriptions orders (+ 0.26 [*P* < 0.001]) and a higher probability of all-cause inpatient (+ 1.4% [*P* = 0.009]), outpatient (+ 6.1% [*P* = 0.006]), or UTI-related outpatient (+ 3.7% [*P* = 0.039]) encounters. Patients with a uUTI and an antibiotic-not-susceptible isolate were significantly more likely to progress to cUTI than those with susceptible isolates (odds ratio: 2.35 [confidence interval: 1.66–3.33; *P* < 0.001]). Over 6 months, patients with not-susceptible versus susceptible isolates had significantly higher all-cause costs (+ $426 [*P* = 0.031]) and UTI-related costs (+ $157 [*P* = 0.034]).

**Conclusions:**

Patients with a uUTI caused by antibiotic-not-susceptible isolates had higher healthcare resource usage, costs, and increased likelihood of progressing to cUTI than those with antibiotic-susceptible isolates.

## Introduction

Uncomplicated urinary tract infections (uUTIs) are one of the most common bacterial infections in the United States (US), with 50–60% of adult women reporting a presumed uUTI in their lifetime [1]. Urinary tract infections (UTIs) are defined as uncomplicated if there are no functional or anatomical anomalies of the urinary tract and no underlying comorbidities or factors associated with increased colonization and decreased efficacy of therapy, which is indicative of a complicated UTI (cUTI) [2].

One of the primary goals of treating a uUTI is to achieve rapid symptom relief and, therefore, a short course of antibiotic treatment is the preferred option [3]. The Infectious Diseases Society of America (IDSA) recommends nitrofurantoin monohydrate, trimethoprim/sulfamethoxazole (TMP-SMX), fosfomycin trometamol, or pivmecillinam as first-line treatments for uUTI [3].

While antibiotics are generally an effective treatment for uUTI, the number of uUTIs caused by antibiotic-resistant bacteria is increasing. A recent study of urine isolated *Escherichia coli* (*E. coli*) from US female patients with UTI found high levels of not-susceptible uropathogens, with key resistance phenotypes increasing between 2011 and 2019 [4]. A 2019 report by the Centers for Disease Control and Prevention (CDC) found that more than 2.8 million cases of infections are caused by antibiotic-resistant pathogens at any anatomic location occur in the US annually, resulting in approximately 35,000 deaths [5]. This shows a marked increase in infection rate and deaths from the equivalent 2013 CDC report (previously 2 million cases of antibiotic-resistant infections, and 23,000 resultant deaths) [6]. This is part of a broader trend of antibiotic resistance across multiple diseases leading to increased mortality [5, 7]. Despite the high incidence of uUTIs, and increasing prevalence of antibiotic resistance, data are lacking on the association between antibiotic resistance and the economic burden of uUTI. While the annual cost associated with uUTI (overall) has previously been estimated at approximately $1.6 billion in the US [8], increases in the rate of antibiotic-resistant pathogens in uUTIs would suggest that costs have escalated in recent years. Previous studies have demonstrated that increased resistance to antibiotics and treatment failure rates result in greater treatment costs compared to susceptible uUTI infections [9]; however, a contemporary estimate is required to understand the current fiscal impact of antibiotic resistance on uUTI resource use and costs.

This study reports a comparison of the economic burden for female patients with uUTI with isolates that were either susceptible or not-susceptible to the initial antibiotic prescription. Using data from a large Mid-Atlantic US integrated delivery network (IDN), this study investigated the relationship between uUTIs caused by not-susceptible uropathogens and healthcare resource use and costs.

## Methods

### Study design

**Fig. 1:**
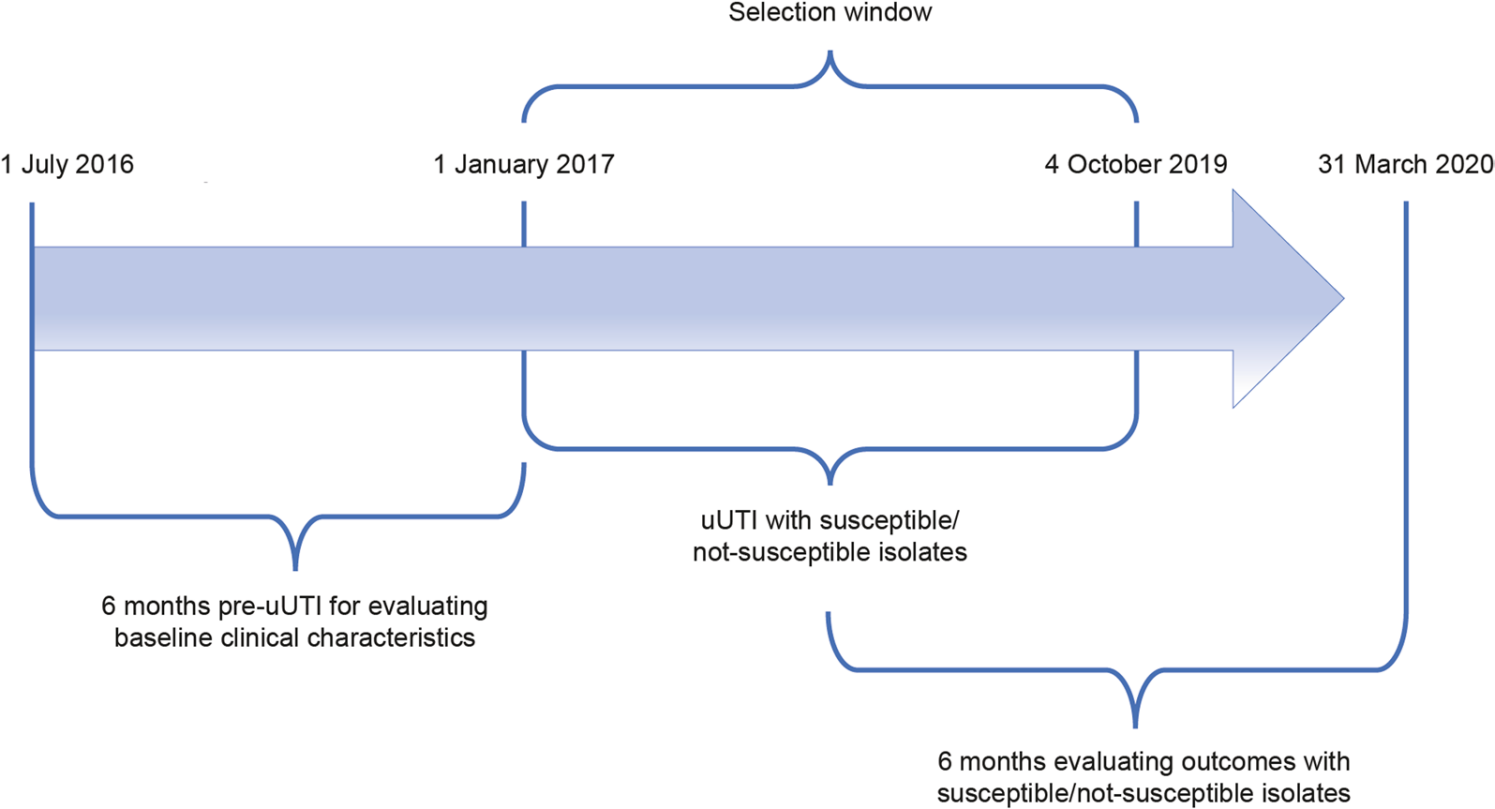
Overview of study design (uUTI patients with susceptible or not-susceptible isolates). *uUTI* uncomplicated urinary tract infection

This was a retrospective cohort study of electronic medical record (EMR) data from an IDN, from 1 July 2016 to 31 March 2020 (Fig. 1). Data were broadly representative of treatment patterns in Virginia, Maryland, and Washington DC. The database contains information on patient diagnosis, prescriptions, procedures, and laboratory values, inpatient visits, and outpatient visits among others. We utilized data from one of the largest IDNs in the Mid-Atlantic region of the US, serving more than 5 million patients.

Antibiotic resistance was reported through antimicrobial susceptibility tests of the isolate in the EMR, rather than at the patient level. The number of isolates was determined and then a decision rule was used to assign a person to a susceptible/not-susceptible status. Sensitive results were categorized as susceptible, whereas resistant and intermediate results were categorized as not-susceptible. For patients with two or more isolates with different susceptibility results (i.e., sensitive results for one isolate and resistant results for another isolate), the individual was classified as being not-susceptible at the person level.

To identify oral antibiotics which are indicated for uUTI, we used the IDSA 2011 guidelines [3] and data published by Brusch et al. [10].

### Patients

Eligible patients were females aged ≥ 12 years, diagnosed with a UTI based on International Classification of Disease, 9th Revision (ICD-9) and ICD-10 coding in the primary or secondary positions, i.e., patients who had ≥ 1 urine culture with 10,000–100,000 colony-forming units (CFU)/mL, had ≥ 1 test to determine antibiotic susceptibility, and received oral antibiotics within ± 5 days of the index uUTI diagnosis to ensure patients had a symptomatic UTI and were receiving empiric therapy with an antibiotic. This treatment window was designed to capture patients with varying periods between diagnosis and treatment and was not designed to represent a change in therapy based on the availability of susceptibility results.

Exclusion criteria were used to further refine the study population to ensure that cases related to cUTI were excluded; a clinical expert was consulted to determine the appropriate exclusion criteria. Individuals were excluded if they were pregnant; had human immunodeficiency virus/acquired immunodeficiency syndrome (HIV/AIDS) with antibiotic use from 6 months to 6 days before the index uUTI date; had a urinary catheter at index UTI event or within 48 h; had symptoms of systemic illness, such as fever (temperature ≥ 38.3 °C), nausea or vomiting, reported or reproduced flank pain at index UTI event or within 48 h of the index UTI event; received intravenous (IV) antibiotics as initial therapy; had renal or urologic abnormalities, immunocompromising conditions, or chronic conditions that lead to systemic infections. Patients with missing laboratory values or utilization measures were also excluded.

### Objectives

The primary objective was to measure the difference in healthcare resource use and associated costs (both all-cause and UTI-related) between patients with susceptible and not-susceptible urine isolates in the 6 months post-index date (inclusive). The primary outcome was related to the EMR encounter data (e.g., hospitalizations, office visits, emergency department [ED] visits, etc.) and the imputed cost associated with each of these.

Secondary objectives included measuring the difference in medical, pharmacy, and antibiotic costs (all-cause and UTI-related) in the 6 months after uUTI between patients with uUTI isolates that were susceptible and not-susceptible. In addition, we assessed the differences in hospital admissions, ED visits, urgent care visits, office visits, and laboratory tests in the 6 months after the index uUTI diagnosis. We also evaluated the difference in probability of uUTI progressing to cUTI between patients who had susceptible or not-susceptible isolates. In terms of disease progression, cUTI was defined as a combination of new fever, nausea, or vomiting, in addition to uUTI symptoms; or receipt of an IV antibiotic 3–28 days after index uUTI. The index date was defined as the first uUTI diagnosis/urine culture with a prescription for an antibiotic with no prior uUTI in the last 28 days.

Since information on prescription fills within the IDN system would be incomplete if the prescription was filled outside the IDN, our analysis relied on medication prescriptions rather than actual use.

The key dependent variables for the analyses were difference in cost and healthcare utilization and the key independent variable was whether an infection involved isolates that were susceptible or not-susceptible. Several other independent variables were included in the analysis as covariates to inform the propensity score matching. These included demographics (age, race, ethnicity), health insurance plan, and Charlson comorbidities (hemiparesis, renal disease, myocardial infarction, chronic pulmonary disease, rheumatic disease).

### Cost calculations

Medical costs were calculated using Medicare fee-for-service rates. However, these rates were then adjusted for payer mix of our data using the relative reimbursement for inpatient stays across payers as reported based on hospital charge and cost-to-charge ratios identified in the Healthcare Cost and Utilization Project [11]. For inpatient costs, we used 2018 100% Medicare limited dataset inpatient and calculated adjusted total paid to 2020 quarter (Q) 1 dollars (using Consumer Price Index for Medical Care) for each diagnosis-related group program. The process for mapping outpatient cost was more heterogeneous. We first calculated the adjusted total paid per Current Procedural Terminology (CPT) code from 5% Medicare outpatient and carrier files. We primarily used outpatient files, but if a CPT code was not found in the outpatient files, then the calculated total paid value from the carrier file was used. For pharmaceutical costs, we used market history to identify wholesale acquisition cost prices from ProspectoRx. For ED costs, we obtained costs from the literature [12, 13] and used the mean of both UTI-related and all-cause visits. All costs were adjusted to 2020 Q1 US dollars ($). Additionally, we constructed winsorized cost outcome variables to minimize the effect of abnormally high utilization patients on our estimates. Categorical costs, such as all-cause drug costs in the follow-up window, were set to the 98th percentile values. In other words, for patients with costs below the 98th percentile, winsorized costs were set to actual cost; those with costs above the 98th percentile had winsorized cost set to 98th percentile cost. The goal of this procedure was to reduce the influence of outlier observations. All-cause services included the cost of all services provided to patients with uUTI in the 6 months after the index uUTI event, while UTI-related costs included the costs of services provided where a uUTI diagnosis code was included in the service event in the encounter file or the service was a uUTI-related lab/culture.

### Statistical analyses

For the primary outcome measure, to evaluate how infection with not-susceptible isolates affected healthcare utilization and costs, propensity score matching was used to align the populations in terms of the following covariates: age; White race; private insurance; inpatient/outpatient clinic/outpatient other/ED visits within 180 days pre-index; and all-cause drug orders within 180 days pre-index. Patients with uUTIs with isolates not-susceptible to ≥ 1 antibiotic were matched with those with susceptible urine isolates using 1:1 nearest neighbor propensity score matching to minimize the difference across the pairs. As cost data are typically right-skewed, we used a generalized linear model (GLM) with a log link and gamma family to account for this skewness in the dependent variable; we split the model into an analysis of the effect of resistance on the extensive margin and intensive margin, individually. Patients retained for analysis had a non-zero predicted probability of being in the case and control group and were retained for analysis only if there were patients in the mirror group with similar propensity scores. The propensity score was calculated using a logistic regression.

Secondary analyses used the same propensity score matching approach as for the primary analysis. The difference in cost outcomes of interest was measured using the same GLM with a log link and gamma family to adjust for patient characteristics. When calculating how antibiotic resistance affected healthcare utilization, a negative binomial model was used to analyze the effect of antibiotic resistance on the following discrete outcome variables: the probability of having at least one all-cause/UTI-related inpatient visit; the probability of having at least one all-cause/UTI-related outpatient visit; or the probability of having at least one all-cause/UTI-related ED visit.

Logistic regression specification was used to estimate the likelihood of progressing to cUTI. Patients were identified as having progressed to cUTI if they had a new symptom for fever, nausea, vomiting, or received a new administration of IV antibiotics between 3 and 28 days post-index uUTI event. Following this, 6-month cost and utilization numbers for patients with uUTI who progressed to cUTI within 28 days of the index uUTI diagnosis were compared with those who did not progress. The same logistic regression models were used to estimate the prediction of the treatment failure outcomes given a patient’s antibiotic resistance status. Logistic regression models were estimated only on the unmatched baseline population of patients who could achieve a given outcome (e.g., the population used in the analysis of the probability of failure of a first-line therapy was restricted to patients who received a first-line therapy as their index antibiotic).

Sensitivity analyses were performed to test the robustness of the primary analysis. In sensitivity analysis #1, patients were required to have both a diagnosis code and positive urine cultures for uUTI inclusion, applying the same 6-month follow-up window as the baseline analysis, to make the population smaller and increase confidence that only patients with uUTI were being analyzed. Sensitivity analysis #2 defined a 30-day post-index uUTI follow-up window that was compared with the 6-month window used to measure all-cause/UTI-related cost outcomes, where previously this was used to measure utilization costs only. Sensitivity analysis #3 used a 12-month post-index uUTI follow-up window to measure utilization and cost of the primary and secondary outcomes, respectively, in order to better capture the long-term effects of infections attributed to antibiotic resistance. In sensitivity analysis #4, the exclusion criteria were simplified and reduced per a previous study [14] to have a less restrictive uUTI study population. Sensitivity analysis #5 was designed to measure the difference in UTI-related and total healthcare resource utilization and costs to compare patients with recurrent uUTIs to patients with one episode of uUTI. In sensitivity analysis #6, patients with concurrent non-uUTI conditions who could be treated with an antibiotic were excluded to ensure infectious conditions with major economic burden were not overinflating costs. Finally, sensitivity analysis #7 was designed to investigate whether the majority of the inappropriate prescribing practices were related to fluoroquinolones (FQs), as their use is discouraged by IDSA guidelines [3]. We repeated the primary analysis but limited the sample to patients receiving FQs as first-line therapy.

## Results

### Patients

**Fig. 2:**
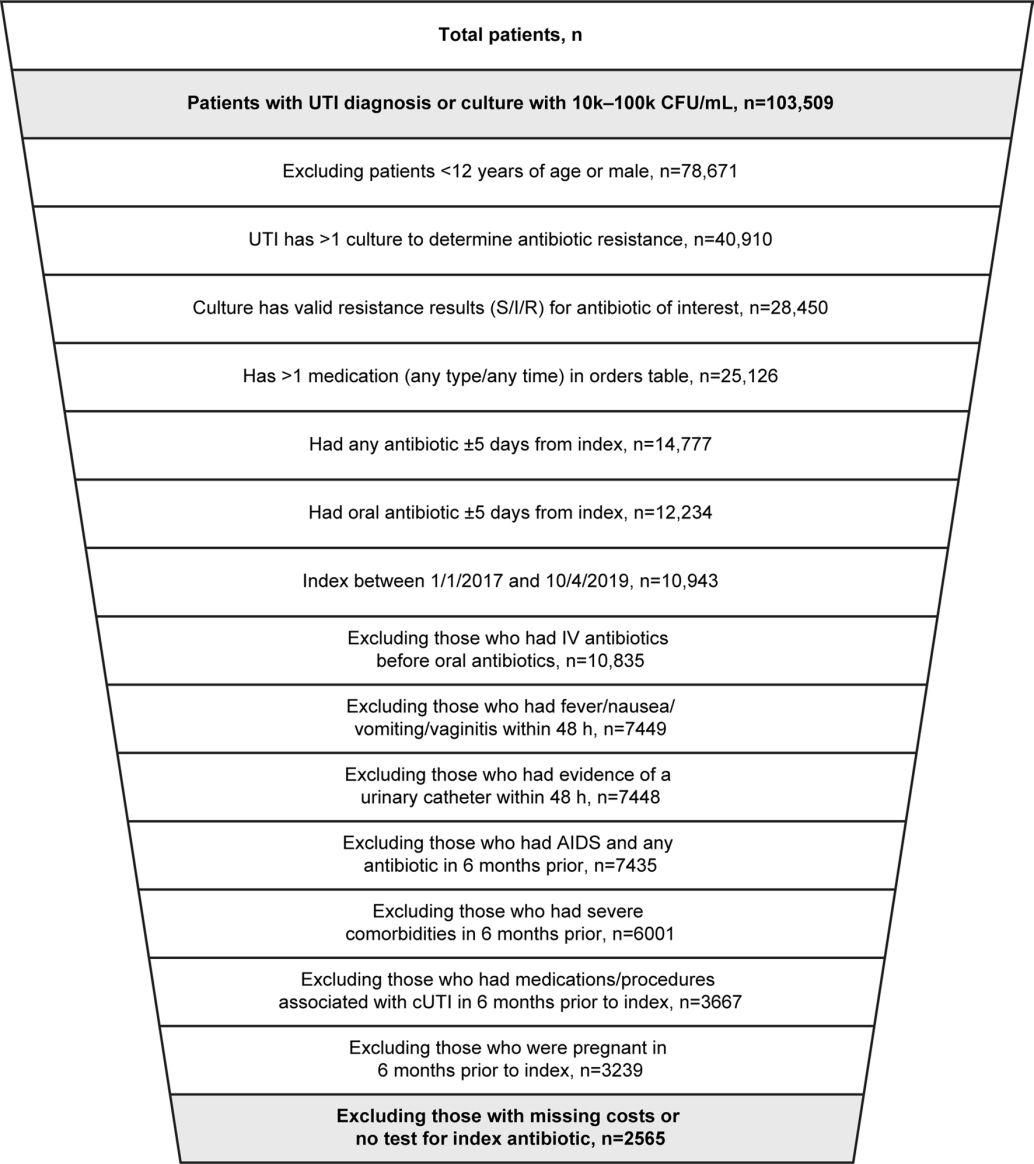
Patients identified with UTI and applied exclusion criteria. *AIDS* acquired immunodeficiency syndrome; *CFU* colony forming units; *cUTI* complicated urinary tract infection; *I* intermediate; *IV* intravenous; *R* resistant; *S* sensitive; *UTI* urinary tract infection

Using the IDN database, 103,509 unique patients had a UTI diagnosis based on ICD-9/10 codes or culture with 10^3^–10^5^ CFU/mL identified. Overall, there were 9479 inpatient admissions and > 1 million outpatient visits. After applying the inclusion and exclusion criteria with consideration of line of therapy, the eligible study population consisted of 2565 patients (Fig. 2). The average age of the patients was 42.8 years for patients who were susceptible compared with 44.4 years among those not-susceptible and 61.1% and 58.2%, respectively, of the sample were White. After 1:1 propensity score matching, the eligible study population comprised 2018 patients. The average age for the susceptible population was 44.0 years of age with 58.6% White compared to 44.1 years of age and 58.4% White for the not-susceptible (Table 1). More broadly, after the propensity score matching, the matched variables were well balanced across the susceptible and not-susceptible cohorts based on standardized mean difference. The most commonly prescribed antibiotic was nitrofurantoin (60.8%), followed by TMP-SMX (19.4%) and ciprofloxacin (14.6%).

**Table 1:** Characteristics of study population by antibiotic-not-susceptible uUTI versus antibiotic-susceptible uUTI during first-line therapy prescribed

Variable	Unmatched (n = 2565)			Matched (n = 2018)		
	Susceptible (n = 1535)	Not-susceptible (n = 1030)	Std. mean difference	Susceptible (n = 1009)	Not-susceptible (n = 1009)	Std. mean difference
**Demographic characteristics**						
Mean age, years	42.83	44.42	0.081	43.95	44.12	0.008
Race, mean proportion of patients						
*White*	0.611	0.582	0.060	0.586	0.584	0.004
*African American*	0.243	0.273	0.068	0.259	0.268	0.020
*Asian*	0.033	0.034	0.004	0.035	0.035	0
*Other race*	0.059	0.057	0.006	0.063	0.058	0.021
*Unknown race/none/declined to answer*	0.054	0.054	0.001	0.057	0.056	0.009
Ethnicity, mean proportion of patients						
*Hispanic*	0.030	0.030	0.001	0.030	0.031	0.006
*Non-Hispanic*	0.872	0.876	0.012	0.879	0.873	0.018
*Other ethnicity*	0.002	0.007	0.073	0.001	0.007	0.095
*Unknown (none/declined to answer)*	0.096	0.087	0.031	0.090	0.089	0.003
**Health insurance, mean proportion of patients**						
Private insurance	0.632	0.585	0.095	0.589	0.595	0.012
Medicare/Medicaid	0.143	0.177	0.091	0.160	0.170	0.029
Other insurance	0.225	0.238	0.031	0.252	0.235	0.039
**Charlson comorbidities, mean numbers**						
Charlson Comorbidity Score	0.053	0.064	0.032	0.053	0.054	0.003
Hemiparesis	0.001	0	0.036	0	0	0
Renal disease	0.001	0	0.036	0	0	0
Myocardial infarction	0	0.001	0.044	0	0.001	0.045
COPD	0.021	0.022	0.006	0.021	0.019	0.014
Rheumatic disease	0.005	0.007	0.021	0.006	0.007	0.012
Peptic ulcer	0.001	0	0.036	0.001	0	0.045
Mild liver disease	0.003	0.004	0.022	0.001	0.004	0.060
Moderate/severe liver disease	0	0.001	0.044	0	0.001	0.045
Dementia	0.006	0.009	0.034	0.008	0.005	0.037
Peripheral vascular disorder	0.003	0.004	0.022	0.003	0.004	0.017
Cerebrovascular disease	0.005	0.003	0.027	0.004	0.003	0.017
Congestive heart failure	0.003	0.001	0.039	0.002	0.001	0.026
**Healthcare resource use (180 days pre-index), mean number of events per patient in the 180 days pre-index**						
Inpatient encounters (all cause)	0.004	0.012	0.077	0.001	0.003	0.045
Outpatient encounters (all cause)	1.024	1.066	0.022	1.014	0.995	0.011
Outpatient clinic encounters (all cause)	0.897	0.975	0.046	0.929	0.912	0.011
Outpatient ambulatory surgery encounters (all cause)	0.01	0.014	0.026	0.012	0.009	0.028
Outpatient other encounters (all cause)	0.117	0.078	0.087	0.073	0.074	0.003
ED encounters (all cause)	0.052	0.084	0.103	0.065	0.066	0.003
Drug orders (all cause)	1.742	2.129	0.068	1.847	1.778	0.016
Any antibiotic use	0.098	0.150	0.158	0.097	0.147	0.152

*COPD* chronic obstructive pulmonary disease; *ED* emergency department; *Std.* standard; *uUTI* uncomplicated urinary tract infection

### Healthcare resource use

**Fig. 3:**
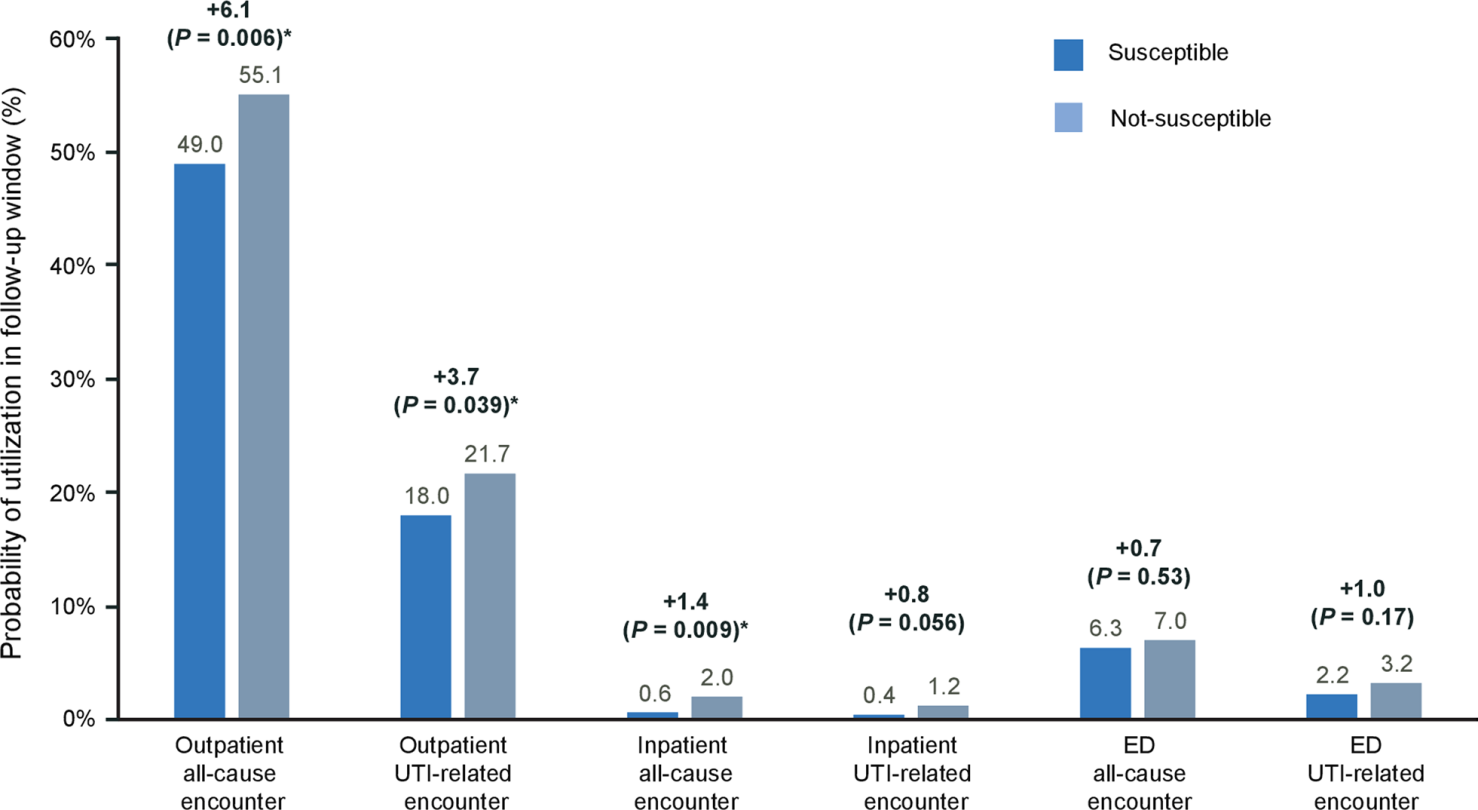
Probability of inpatient, outpatient, and emergency department visits during follow-up in the 6 months or 180 days post-index. Susceptible/not-susceptible refers to the initial antibiotic prescribed. *Statistically significant value (*P* < 0.05). *ED* emergency department; *UTI* urinary tract infection

In the 6 months post-index uUTI event, patients with not-susceptible isolates had significantly higher numbers of prescriptions (+ 1.41 [*P* = 0.001]) and UTI-specific prescriptions (+ 0.26 [*P* < 0.001]) versus patients with susceptible isolates. They also had a higher probability of all-cause outpatient (+ 6.1% [*P* = 0.006]), UTI-related outpatient (+ 3.7% [*P* = 0.039]), or all-cause inpatient (+ 1.4% [*P* = 0.009]) visits (Fig. 3).

### Progression to cUTI

**Fig. 4:**
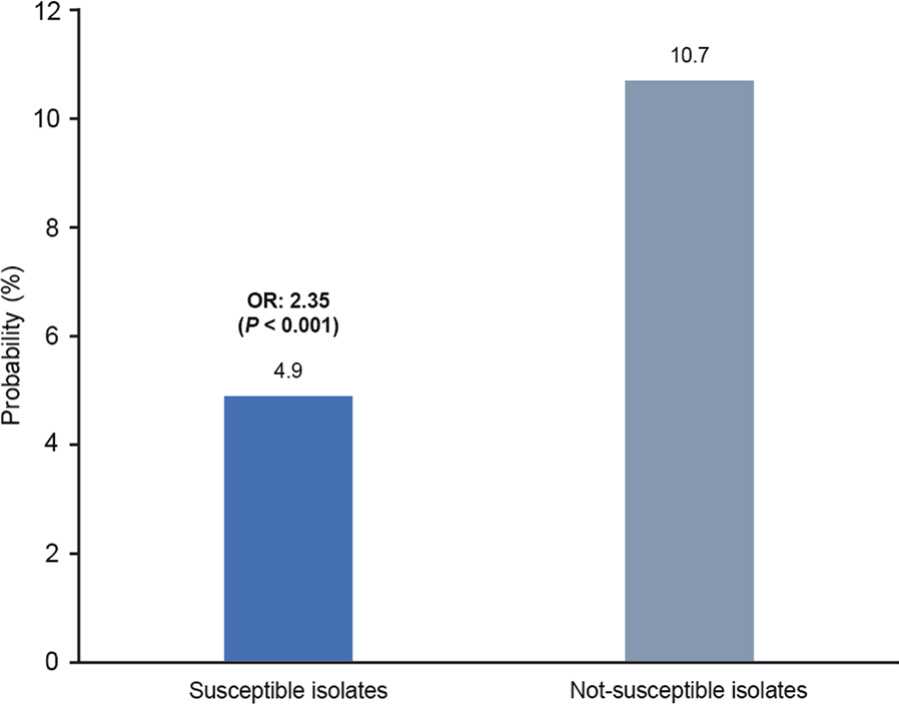
Probability of uUTI–cUTI progression from uncomplicated to complicated UTI among patients with uUTI the 6 months or 180 days post-index. CI: 1.66–3.33. J Shafrin. Progression of an Uncomplicated Urinary Tract Infection Among Female Patients with Susceptible and Non-Susceptible Urine Isolates: Findings from an Integrated Delivery Network. [Paper/Poster/Talk] presented at IDWeek; September 29–October 3, 2021; Virtual Event. https://idweek.org. *CI* confidence interval; *cUTI* complicated urinary tract infection; *OR* odds ratio; *UTI* urinary tract infection; *uUTI* uncomplicated urinary tract infection

The predicted probability of disease progression to cUTI was more than doubled for patients with not-susceptible isolates versus those with susceptible isolates (10.7% vs. 4.9%; odds ratio: 2.35; confidence interval: 1.66–3.33; *P* < 0.001) (Fig. 4).

### All-cause and UTI-related costs

**Fig. 5:**
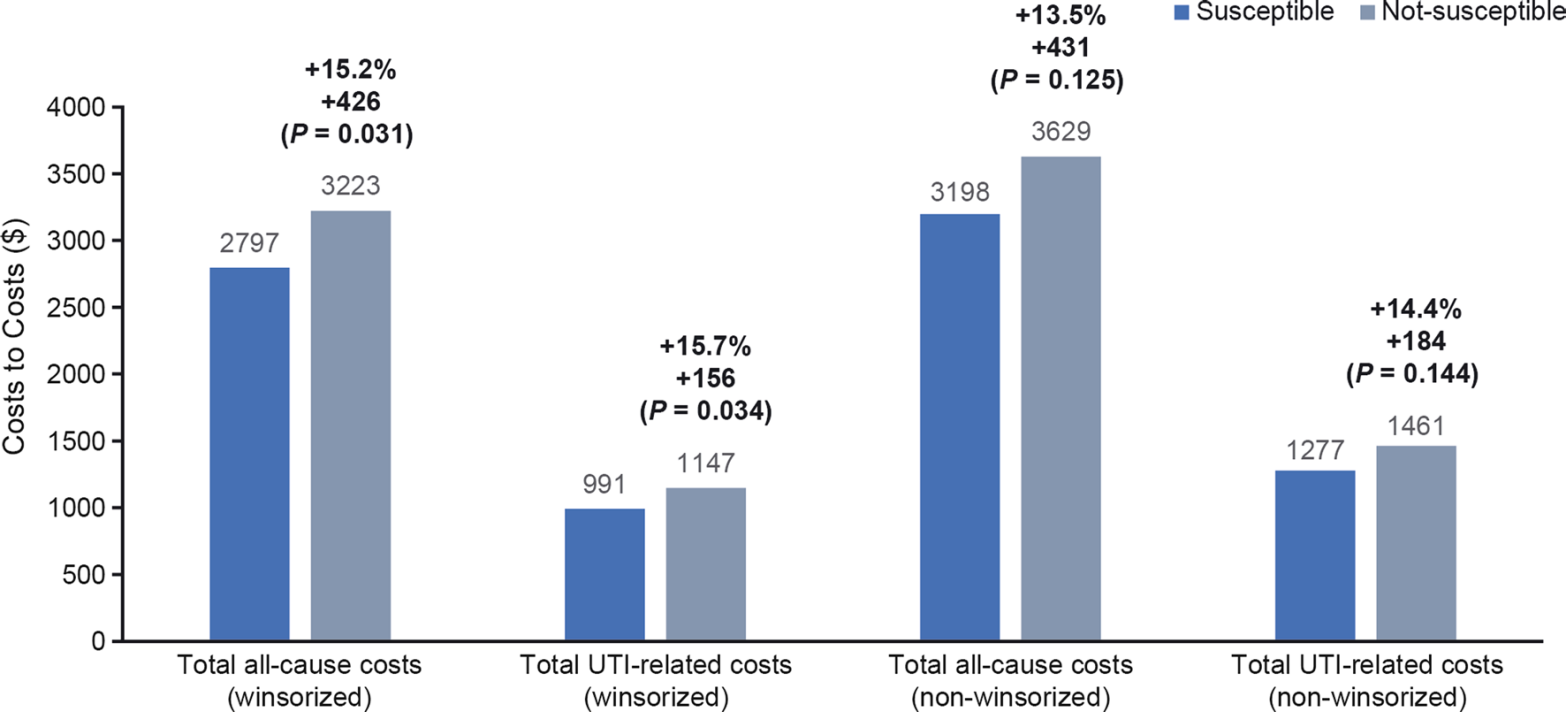
Winsorized and non-winsorized total all-cause/UTI-related costs in the 6 months or 180 days post-index. *UTI* urinary tract infection

The total all-cause costs (winsorized) were estimated at $2797 versus $3223 (*P* = 0.031), for uUTIs with susceptible and not-susceptible isolates, respectively, in the 6 months or 180 days post-index (Fig. 5). Including the index encounter, total UTI-related costs (winsorized) were estimated at $991 versus $1147 (*P* = 0.034), for uUTIs with susceptible and not-susceptible isolates, respectively. Although not statistically significant, non-winsorized all-cause and UTI-related costs were higher for patients with not-susceptible isolates compared to those with susceptible isolates ($3629 vs. $3198, *P* = 0.125; $1461 vs. $1277, *P* = 0.144, respectively) (Fig. 5).

### Medical, pharmacy, and antibiotic costs

**Fig. 6:**
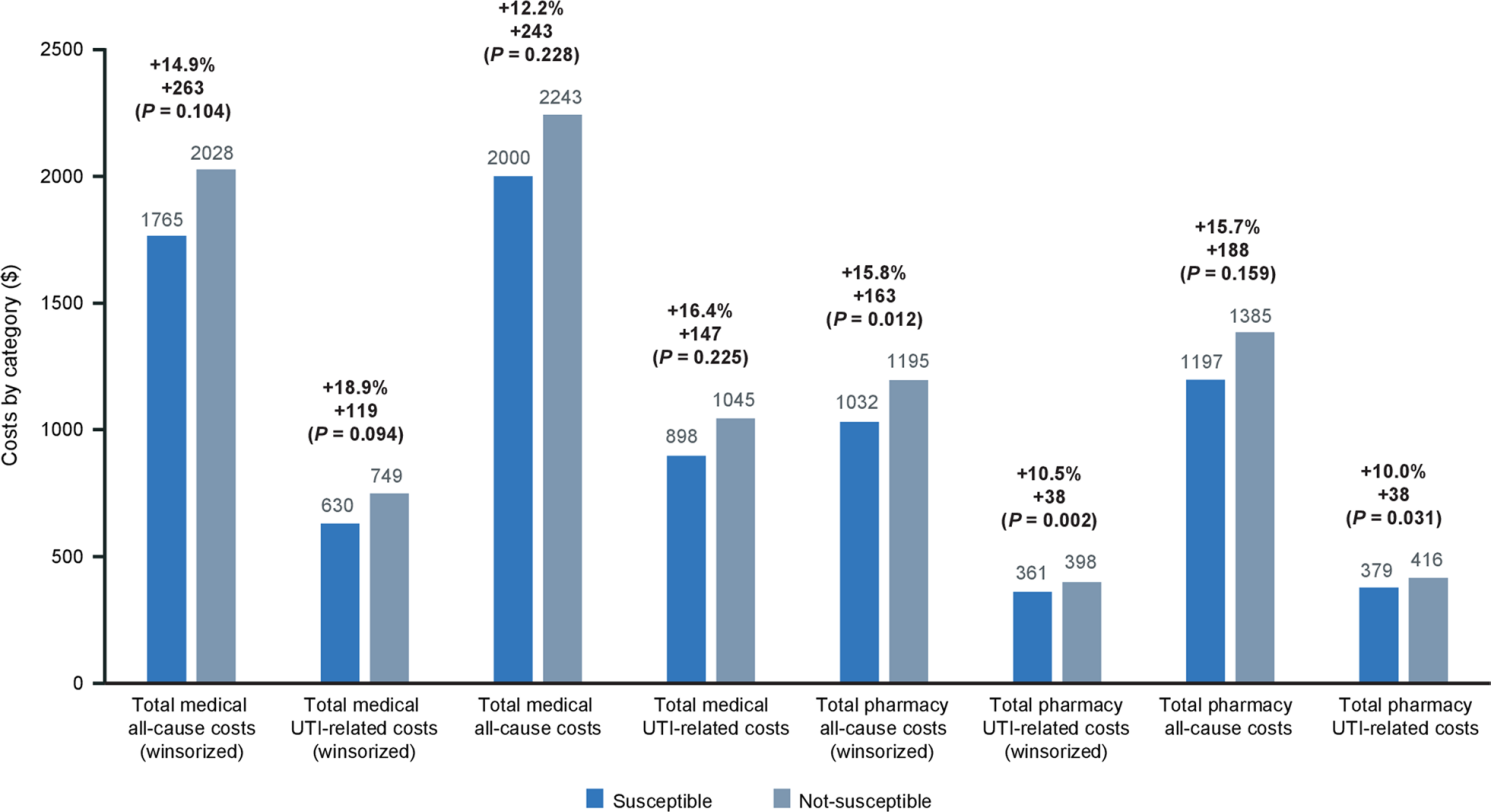
Winsorized and non-winsorized total all-cause/UTI-related costs by medical and pharmacy categories in the 6 months or 180 days post-index. *UTI* urinary tract infection

In the 6 months after uUTI diagnosis, in female patients with susceptible versus not-susceptible isolates, all costs were numerically higher in the not-susceptible group. The differences were statistically significant for total all-cause (winsorized) pharmacy costs (+ $163; *P* = 0.012) and total UTI-related (winsorized) pharmacy costs (+ $38; *P* = 0.002) (Fig. 6). Very similar results were observed from the models of un-winsorized costs (Fig. 6). The effect of not-susceptible isolates on total medical costs (all-cause and UTI-related) was not statistically significant (Fig. 6) but costs were numerically higher for infections with not-susceptible isolates than susceptible isolates. With not-susceptible isolates, medical costs (winsorized) were higher by $263 (*P* = 0.104) and $119 (*P* = 0.094) on average for all-cause and UTI-related spending, respectively.

### Sensitivity analyses

Stricter exclusion criteria were applied in sensitivity analysis #1, reducing the eligible group to 1672 patients (Table 2). The difference in winsorized all-cause costs and winsorized UTI-related costs remained significant for patients with not-susceptible versus susceptible isolates. In the baseline population, non-winsorized all-cause costs and UTI-related costs were not significant but under these stricter exclusion criteria were significant (Table 2). Overall, 3063 patients were eligible for sensitivity analysis #2, with a shorter 30-day follow-up period, and 2157 patients were eligible for sensitivity analysis #3, with a longer 360-day follow-up period (Table 2). In both analyses, non-significant differences were observed across all cost categories between patients with not-susceptible and susceptible isolates (Table 2). Looser exclusion criteria were applied in sensitivity analysis #4, which did not affect the number of eligible patients (n = 2565), resulting in the measured difference across all cost categories remaining almost completely unchanged (Table 2). Sensitivity analysis #5 was not carried out due to insufficient numbers of patients with recurrent uUTI (n < 10). Infectious patients were excluded in sensitivity analysis #6, resulting in a smaller population (n = 2428). The difference in cost outcomes was fairly consistent with the baseline analysis, except for winsorized UTI-related costs, where the difference in costs between antibiotic-resistant and antibiotic-sensitive patients decreased, although this result was not statistically significant (Table 2). Sensitivity analysis #7 only involved patients receiving FQs (n = 398). All cost outcomes were non-significantly higher compared to the baseline analyses (Table 2). Probability of progression to cUTI remained significant across all sensitivity analyses except sensitivity analysis #7 (patients receiving FQs-only) (Table 2).

**Table 2:** Sensitivity analyses summary (uUTI patients with susceptible or not-susceptible isolates)

Sensitivity analysis	Sample size	Difference [not-susceptible − susceptible] (*P*-value)									
		Probability of progressing to cUTI		All-cause costs		UTI-related costs		All-cause costs (winsorized)		UTI-related costs (winsorized)	
		Difference	*P*-value	Difference	*P*-value	Difference	*P*-value	Difference	*P*-value	Difference	*P*-value
Baseline	1009	+ 0.060	< 0.001*	+ 430.9	0.125	+ 184.3	0.144	+ 425.7	0.031*	+ 156.8	0.034*
#1 (strict exclusion)	661	+ 0.039	0.009*	+ 797.7	0.014*	+ 304.6	0.043*	+ 653.5	0.002*	+ 217.5	0.028*
#2 (30-day follow-up)	1200	+ 0.057	< 0.001*	− 12.85	0.910	+ 17.57	0.877	+ 36.00	0.614	+ 22.65	0.555
#3 (360-day follow-up)	834	+ 0.058	< 0.001*	+ 412.2	0.285	+ 126.7	0.409	+ 301.9	0.256	+ 65.25	0.478
#4 (loose exclusion)	1009	+ 0.060	< 0.001*	+ 430.9	0.125	+ 184.3	0.144	+ 425.7	0.031*	+ 156.8	0.034*
#6 (infectious exclusion)	955	+ 0.058	< 0.001*	+ 511.7	0.060	+ 206.5	0.085	+ 471.7	0.012*	+ 73.15	0.139
#7 (FQs-only)	166	+ 0.036	0.387	+ 605.8	0.316	+ 186.3	0.471	+ 484.9	0.307	+ 196.0	0.369

J Shafrin. Progression of an Uncomplicated Urinary Tract Infection Among Female Patients with Susceptible and Non-Susceptible Urine Isolates: Findings from an Integrated Delivery Network. [Paper/Poster/Talk] presented at IDWeek; September 29–October 3, 2021; Virtual Event. https://idweek.org

^*^Statistically significant (*P*-value < 0.05)

*cUTI* complicated urinary tract infection; *FQ* fluoroquinolone; *UTI* urinary tract infection; *uUTI* uncomplicated urinary tract infection

## Discussion

This retrospective cohort study analyzing data from a large IDN in the Mid-Atlantic US region evaluated multiple aspects of the economic costs of antibiotic resistance among patients with uUTI. To our knowledge, no other published studies have examined these aspects using IDN data in the US. The study population was identified after applying UTI inclusion criteria and cUTI exclusion criteria, such as: recent IV antibiotic treatment; experience of fever, nausea, vomiting, and vaginitis; evidence of a urinary catheter within 48 h; had HIV/AIDS; severe comorbidities within the past 6 months; those with medications/procedures associated with cUTI within the past 6 months; pregnancy; and missing costs or no susceptibility test for index antibiotic (Fig. 2). Patients with not-susceptible isolates had higher all-cause costs and UTI-related costs in the 6 months after a uUTI diagnosis in comparison with patients who had susceptible isolates. Patients with not-susceptible isolates also had significantly greater pharmacy costs compared with patients with susceptible isolates, whereas the effects on medical cost outcomes were not statistically significant. Interestingly, patients with not-susceptible isolates were also more than twice as likely to progress from uUTI to cUTI. There are few studies designed to evaluate the probability of uUTI with not-susceptible isolates transitioning into cUTI. Our results showed that patients with uUTI caused by isolates not-susceptible to ≥ 1 antibiotic were more likely to progress to cUTI than patients with susceptible isolates.

Our results expand upon a similar study with data from the United Kingdom by Alam et al. 2009, which evaluated the costs of treating patients with antibiotic-resistant *E. coli* UTI compared to patients with sensitive *E. coli* [15]. This observational study found that patients with *E. coli* resistant to ≥ 1 antibiotic had significantly higher total costs compared with those with sensitive *E. coli*, even after controlling for age, gender, previous bladder operation, comorbidity, and previous catheterization. This study differed from ours in that it included patients with cUTI as well as uUTI, so constituted a population with more severe disease, and used a much older data source.

In another study comparing the costs of not-susceptible versus susceptible isolates in community-acquired UTI [16], the cost of care or hospital costs ($10,741 vs. $7083; *P* = 0.02) and length of stay (6 vs. 4 days; *P* = 0.02) among extended-spectrum β-lactamase-producing *E. coli* and *Klebsiella* species (ESBL-EK) UTI was 1.5 times greater when compared to non-ESBL-EK UTI [16]. These cost estimates reported by MacVane et al. are, however, higher than in our study [16]. This may be due to differing inclusion criteria between the two studies; MacVane et al. assessed outcomes in patients with UTIs admitted to hospital, compared with our study, which had an outpatient population. As such, MacVane et al. did not look specifically at uUTI in their study. The results of the current study also align with other studies in the literature where antimicrobial resistance has been associated with higher treatment costs [9].

It should be noted that the definitions of susceptible, intermediate, and resistant by isolate type can differ by region (e.g., European Committee on Antimicrobial Susceptibility Testing [EUCAST] vs. Clinical & Laboratory Standards Institute [CLSI]) and across studies. For example, according to EUCAST guidelines [17], Enterobacterales isolates with intermediate susceptibility are categorized as susceptible, whereas in the US, per CLSI guidelines [18], isolates with intermediate susceptibility results are categorized as not-susceptible. Additionally, a 2019 Dutch study [19] reported susceptibility rates for amoxicillin, trimethoprim, and ciprofloxacin are 66, 79, and 94%, respectively. Thus, our reported susceptibility rates are in line with this study.

From the wider literature, several factors are known to drive additional healthcare costs. For example, the increased incidence of hospitalizations for UTIs has led to higher costs, far more than additional prescriptions [20–22]. In our study, we observed that more patients with not-susceptible urinary isolates required hospital clinic/ED visits and progressed to cUTI than those with susceptible urinary isolates. However, it should be noted that our data set includes prescriptions that were written, not necessarily filled, and we did not have information on individual patient treatment pathways employed by the health system at that time.

Findings from our study may have clinical implications and highlight the need for the development of new antibiotics to combat antibiotic-resistant isolates. uUTIs are generally treated in outpatient settings with antibiotic prescriptions based on treatment guidelines and patient symptoms alone [23]. By treating these patients without specific knowledge of the pathogen or antibiotic susceptibility, there is a possibility that patients might be prescribed an antibiotic therapy to which their isolate is not-susceptible, leading to a higher probability of treatment failure and subsequent infections with antibiotic-resistant uropathogens [24]. As the prevalence of antibiotic resistance has significantly increased in the US among community-acquired uUTIs [4, 25], it is crucial to shift away from prescribing purely empirically to avoid adverse consequences, including increased antibiotic resistance and disease progression. Empiric treatment can be improved by using regional antibiotic resistance rates and individual patient risk factors for antibiotic resistance (taking into account prior history) to guide treatment decisions. To support this approach, there is a need for development of new treatment guidelines, patient risk-based recommendations, and rapid point-of-care diagnostic tests to detect resistance among the most common uUTI isolates such as *E. coli, Staphylococcus saprophyticus,* and *Klebsiella pneumoniae*, as well as updated uUTI management strategies.

A strength of this study is the use of recent EMR data from a large US IDN, where the network has a diverse population both in terms of population density (i.e., urban, suburban, and rural areas), as well as its racial and socioeconomic composition. We also evaluated multiple aspects of the economic costs of antibiotic resistance among uUTI patients. Finally, our use of laboratory data allowed us to directly observe whether patients had infections with isolates susceptible versus resistant to the antibiotics prescribed.

Limitations of the study include that it was conducted using a single IDN, meaning that the results may not necessarily be generalizable beyond the Mid-Atlantic US. In addition, uUTI diagnosis may have been imperfect in that urine cultures may not have been performed randomly, which may have resulted in underreporting of the population or biased sampling; the use of ICD-9 or ICD-10 codes could also have overestimated or underestimated the diagnosis of uUTI since it is dependent on the hospital coder. However, in this study, diagnostic data were confirmed through one of the previously described sensitivity analyses. The treatment of uUTI was empirical and therefore the final study cohort was limited to those with culture based on the objectives. The exclusion of patients without culture may have biased the study towards the inclusion of patients with recurrent UTI and/or higher rates of not-susceptibility as compared to the general uUTI population; however, it is not possible to assess healthcare resource use and associated costs by patients with not-susceptible isolates if culture data are not available. Additionally, the identification of a uUTI diagnosis is only as accurate as the detail provided by physicians in the EMR. Furthermore, medical records could also contain misclassification of uUTI and cUTI if the symptoms are not noted correctly in the EMR. Another limitation is that the study required patients with uUTI, positive culture and treatment; since many patients are treated empirically, many uUTI patients were therefore not eligible for study inclusion. It is known that approximately 20–30% of patients with a uUTI will have a negative urine culture which is not captured in the study population [26]. The inclusion requirement of a positive culture and antibacterial treatment could potentially have led to selection bias or the inclusion of more patients with recurrent UTI (whose isolates are typically cultured more), potential inclusion of a higher proportion of patients with resistant isolates and thus an overestimation of healthcare resource use and costs compared to the general uUTI population which includes those not cultured and those with a negative index culture.

Over-the-counter medications frequently used to treat uUTI—such as painkillers—were not captured in the EMR data, nor was whether the individuals moved or changed their regular provider. Although we did consider requiring individuals to have visits after the follow-up period, doing so would have biased the sample towards higher-cost patients. Because patients may change providers during the follow-up period, our current approach should be considered a conservative estimate of the impact of antibiotic resistance among patients with uUTI. Lastly, inpatient costs were calculated using Medicare rates, and privately insured patients’ costs were adjusted based on the ratio or cost-to-charge ratio in Selden (2020) [11]. On one hand, this approach makes the cost data less idiosyncratic to rates of the IDN used. On the other hand, if variability in reimbursement for non-hospital services differs in ways dissimilar from the hospital services recoding in the Selden publication [11], the cost information may over- or underestimate costs for non-Medicare patients.

## Conclusions

Patients with uUTI caused by antibiotic-not-susceptible urinary isolates had higher all-cause costs and UTI-related costs in the 6 months post-index in comparison to patients with antibiotic-susceptible isolates. We found that patients with not-susceptible isolates had significantly greater pharmacy costs compared with patients with susceptible isolates. Furthermore, patients with not-susceptible isolates were significantly more likely to have outpatient and all-cause inpatient visits than those with susceptible isolates. Lastly, patients in our study with uUTI caused by not-susceptible isolates were significantly more likely to progress to cUTI than those with susceptible isolates.

## Data Availability

The authors confirm that the data supporting the findings of this study are available within the article and its supplementary materials.

## Abbreviations

AIDS: Acquired immunodeficiency syndrome
CDC: Centers for Disease Control and Prevention
CFU: Colony-forming units
CI: Confidence interval
CLSI: Clinical & Laboratory Standards Institute
COPD: Chronic obstructive pulmonary disease
CPT: Current Procedural Terminology
cUTI: Complicated urinary tract infection
ED: Emergency department
EMR: Electronic medical record
ESBL-EK: Extended-spectrum β-lactamase-producing *E. coli* and *Klebsiella* species
EUCAST: European Committee on Antimicrobial Susceptibility Testing
FQ: Fluoroquinolone
GLM: Generalized linear model
HIV: Human immunodeficiency virus
ICD: International Classification of Diseases
IDN: Integrated delivery network
IDSA: Infectious Diseases Society of America
IV: Intravenous
OR: Odds ratio
Q: Quarter
S/I/R: Susceptible/intermediate/resistant
Std.: Standard
TMP-SMX: Trimethoprim/sulfamethoxazole
US: United States
UTI: Urinary tract infection
uUTI: Uncomplicated urinary tract infection
